# Development and validation of a self-reported questionnaire to assess occupational balance in parents of preterm infants

**DOI:** 10.1371/journal.pone.0259648

**Published:** 2021-11-15

**Authors:** Mona Dür, Anna Röschel, Christiane Oberleitner-Leeb, Verena Herrmanns, Elisabeth Pichler-Stachl, Barbara Mattner, Silvia-Desiree Pernter, Martin Wald, Berndt Urlesberger, Herbert Kurz, Thomas Frischer, Karl Zwiauer, Angelika Berger

**Affiliations:** 1 Department of Pediatrics and Adolescent Medicine, Division of Neonatology, Pediatric Intensive Care and Neuropediatrics, Comprehensive Center for Pediatrics, Medical University of Vienna, Vienna, Austria; 2 Department of Health Sciences, IMC University of Applied Sciences Krems, Krems, Austria; 3 Duervation, Krems, Austria; 4 Department of Pediatrics with Neonatology, St. Josef Hospital Vienna, Vienna, Austria; 5 Department for Pediatrics and Adolescent Medicine, Division of Neonatology, Medical University of Graz, Graz, Austria; 6 Department of Pediatrics and Adolescent Medicine, Division of Neonatology, Pediatric Intensive Care, Wilhelminen Hospital, Vienna, Austria; 7 Department of Pediatrics and Adolescent Medicine, Division of Neonatology, University Hospital Salzburg, Salzburg, Austria; 8 Department of Pediatrics and Adolescent Medicine, Social Medical Center East–Danube Hospital Vienna, Vienna, Austria; 9 Sigmund Freud University, Vienna, Austria; 10 University Hospital for Pediatrics and Adolescent Medicine, University Hospital Sankt Pölten, St. Pölten—Karl Landsteiner University for Health Sciences, Krems, Austria; Sunway University, MALAYSIA

## Abstract

**Background:**

Parents’ meaningful activities (occupations) and occupational balance are relevant to neonatal care. Valid and reliable self-reported measurement instruments are needed to assess parents’ occupational balance and to evaluate occupational balance interventions in neonatal care. The aims of this study were to develop a self-reported questionnaire on occupational balance in informal caregivers (OBI-Care) and to examine its measurement properties including construct validity and internal consistency.

**Methods and findings:**

A mixed method multicenter study design was employed. Items of the OBI-Care were created with parents of preterm infants based on qualitative research methods. Measurement properties were analyzed with quantitative data of parents of preterm infants. Construct validity was assessed by determining dimensionality, overall and item fit to a Rasch model, differential item functioning and threshold ordering. Internal consistency was examined by determining inter-item and item-total correlations, Cronbach’s alpha and Rasch’s person separation index. Fourteen parents participated in item creation. Measurement properties were explored in data of 304 parents. Twenty-two items, summarized in three subscales were compiled to the OBI-Care. Items showed an overall fit and except one item, an item fit to the Rasch model. There was no evidence of differential item functioning and all items displayed ordered thresholds. Each subscale had good values of person separation indices and Cronbach’s alpha.

**Conclusions:**

The OBI-Care demonstrates construct validity and internal consistency and is thus a suitable measurement instrument to assess occupational balance of parents of preterm infants in neonatal care. OBI-Care is generic and can be applied in various health care settings.

## Introduction

Informal caregivers provide unpaid care for family members or friends with health issues or disabilities. Thus, parents caring for children with impairments or a disease, such as parents of preterm infants are defined as informal caregivers too [1–3]. Approximately fifteen million (11%) infants worldwide are born preterm (< 37 completed weeks of gestation) every year [4]. Among these, an estimated million die due to complications of prematurity [5–7]. Additionally, preterm infants run a high risk of neurodevelopmental disabilities [8–14], which both have a strong impact on parents’ health and well-being. Studies showed that informal caregivers, such as parents of preterm infants experience a high caregiver burden and restricted subjective physical and mental health [15–18]. A child’s development can be adversely affected by parental physical and mental health [15, 19]. The main targets of neonatal intensive care are survival, prevention of complications of prematurity and improvement of long-term outcomes. Parents of preterm infants also start to focus exclusively on activities that serve the survival and improvement of their child’s health and well-being and neglect their own needs. As a consequence, their own health and well-being is negatively affected [20, 21].

Moreover, neonatal intensive care includes a strong involvement of parents [2, 22, 23]. For example, parents are encouraged to engage in feeding activities, skin-to-skin care, and kangarooing [22, 24–27]. As a result, parents’ daily routine is mostly determined by activities of parenting and caregiving, which causes a restriction of other meaningful activities [28–31]. Deviations in the engagement of meaningful activities cause changes of occupational balance, defined as a satisfying amount and variety of meaningful activities [32]. There is evidence that informal caregiving leads to limited occupational balance [29, 31, 33] and a need for interventions to support informal caregivers’ occupational balance [33, 34]. Furthermore, occupational balance was found to be associated with health, quality of life and well-being [32, 35–38]. For example, Park et al. reported direct effects of occupational balance on health, well-being and quality of life [38]. Thus, enhancing one’s subjective health, well-being, and quality of life might improve one’s occupational balance [38].

Additionally, associations between parental and infantile health and well-being were proven [24–26]. Moreover, parents’ occupational balance was found to be related to infantile health. For instance, parents’ occupational balance was related to weight loss of their obese child [39]. Inferring, parents’ occupational balance might be associated with the health and well-being of preterm infants too, and therefore might be clinically relevant in neonatal intensive care [40]. Thus, it is important to address and strengthen parents’ occupational balance in neonatal intensive care [40, 41]. To address parents’ occupational balance, health professionals in the neonatal intensive care unit (NICU), such as occupational therapists or psychologists need valid and reliable occupational balance measurement instruments, such as a self-reported questionnaire developed for or validated in neonatal intensive care [42]. Since occupational balance is subjective, its self-evaluation by the individual is required. Self-reported measurement instruments, such as self-reported questionnaires, are commonly used for self-evaluation [42].

The valid and reliable use of a self-reported questionnaire is determined by satisfying measurement properties. However, to our knowledge, no self-reported measurement instrument on occupational balance exists which was developed for or validated in neonatal intensive care [43]. Additionally, no measurement instrument specifically developed or validated to assess occupational balance of any other informal caregivers exits [43]. The aims of this study were to develop a self-reported questionnaire on occupational balance in informal caregivers (OBI-Care) and to examine its measurement properties including construct validity and internal consistency.

## Materials and methods

The development of self-reported outcome measures is complex, requires the use of different research methods and should include the involvement of the persons affected [44]. Therefore, a mixed methods design was applied for the OBI-Care development process, including a qualitative approach for the creation of a set of items to assess occupational balance in informal caregivers, and a quantitative approach for the exploration of its measurement properties and a final compilation of the questionnaire [45, 46]. The study was part of a larger project on occupational balance [40].

### Recruitment

A set of items was created in collaboration with parents of preterm infants from the NICU at the Medical University of Vienna in November and December 2015. Parents of preterm infants treated at the Medical University of Vienna that met inclusion criteria were informed about the study and asked for participation and their written and verbal consent by the first author (MD). Measurement properties were examined in the data of further parents of preterm infants treated at NICUs at the Medical University of Vienna, Wilhelminen Hospital, Social Medical Center East—Danube Hospital, University Hospital St. Pölten, University Hospital Salzburg and the Medical University of Graz from 2016 to 2018. To examine measurement properties of the OBI-Care, parents of preterm infants from one of the participating centers who met the inclusion criteria were informed about study procedures and were asked to give written and verbal informed consent. These participants were recruited by nurses, occupational therapists (including the first author [MD]), pediatricians, psychologists, speech therapists and music therapists of the NICU. Inclusion criteria for item creation and examination of measurement properties were parents of preterm infants born alive prior to complete 37 weeks of gestation with a very low birth weight of ≤ 1500 grams. Parents with insufficient German reading and speaking skills and with psychiatric or neuro-motor diseases were excluded. Another exclusion criterion was the decease of their preterm infant(s).

The sample size for the item creation was defined according to Coons et al., who suggest at least five to ten participants for cognitive debriefing [47]. Considering the examination of measurement properties, the sample size was based on recommendations for analyses with a Rasch model [48]. These analyses require ten observations (persons) in each category for each item whereby observations do not have to be unique cases [48].

### Creation of a set of items

The creation of the set of items was based on a phenomenological approach, allowing an insight into actual experiences and views of personally affected persons [49], such as parents of preterm infants who experienced changes in occupational balance. Prior to the current study, components of occupational balance that were meaningful to parents of preterm infants were identified in the analysis of focus group interviews (unpublished work). Based on the identified components initial OBI-Care items were created by the first author (MD) in collaboration with two mothers and two fathers of preterm infants. For each identified component, several specific and generic items were formulated. Response categories were formulated following standard procedures [42]. Within two sessions of two hours each, a first version of draft items was created together with the parents. Therefore, parents received basic information on questionnaire development with a special focus on item creation (MD). Parents were then asked to formulate two to three items for each component. Based on the consensus between parents and the first author (MD), individual items were discussed, eventually rephrased, and their use in the first draft was determined.

Subsequently, cognitive debriefing [37, 50] with further parents of preterm infants and with a panel of experts, consisting of different health professionals, was conducted to get feedback on comprehensibility and applicability of the items. Cognitive debriefing followed standard procedures as defined by Willis [50], such as the application of thinking-aloud and verbal probing procedures. Notes were taken during the debriefing sessions. According to the parents’ and health professionals’ feedback some items were rephrased. Thereby the items’ face validity was ensured [42]. The first author (MD), who conducted cognitive debriefing, is trained, and experienced in the use of qualitative research methods, such as focus group interviews and cognitive debriefing. Basing the items upon components which were meaningful to parents of preterm infants, co-creating the items together and rephrasing them based on the results of cognitive interviews with parents of preterm infants and experts contributed to the reduction of researcher bias [51] and the questionnaire’s content validity [46].

### Exploration of measurement properties

Participants were asked to fill in several self-reported questionnaires [52–57] including the OBI-Care. After data collection, data were entered into a Statistical Package for the Social Sciences (SPSS [58]) data file and read into the computer program “Rasch Unidimensional Measurement Model 2030” (RUMM2030 [59]). Data of participants with more than ten missing items were excluded. The following measurement properties of the OBI-Care were explored: construct validity and internal consistency. Construct validity refers to the accordance among expected scores and existing knowledge and hypotheses of the construct to be measured. Internal consistency is defined as the degree of interrelatedness among a scale’s items and thus provides information on the extent to which the items measure the same construct [42, 46].

As part of construct validity, dimensionality of the first set of items was evaluated by conducting t-tests with RUMM2030 and factor analysis with SPSS. Less than 5% of the t-tests located outside the acceptable significance range (-1.96 to +1.96) indicate unidimensionality of the scale [60–62]. Due to the identification of multidimensionality of the measurement instrument, there was a need for the creation of subscales.

Construct validity was examined with various analyses with a Rasch model [45, 60]. An initial inspection of item fit and overall fit to the Rasch model was conducted. For this purpose, item fit residual statistics and item-trait interaction chi-square statistics were examined. Non-significant fit residuals within the range of -2.5 and +2.5 indicate an item fit, non-significant item-trait interaction chi-square values an overall fit. Total chi-square probability values should be higher than 0.05 and mean item fit values close to zero with a standard deviation close to one. Furthermore, differential item functioning (DIF) was explored. A significant DIF indicates that different groups within a sample respond in a different way to an individual item despite equal levels of ability. Potential DIF of the parents’ sex and age (above and below the median) as well as the preterm infants’ sex, gestational age and birth weight were calculated. Moreover, the ordering and distribution of response thresholds for each item were assessed by exploring category and threshold probability curves. Disordered thresholds indicate that the response categories do not operate as intended [61, 63–67]. Items that did not fit the Rasch model were deleted. Significance levels for all analyses were set at α = 0.05. Bonferroni adjustment for multiple testing was used [68]. These methods were applied iteratively until a valid and reliable set of items, summarized in subscales was identified.

Internal consistency was assessed with different correlation statistics following current standards [42]. Inter-Item correlations, item-total correlations and Cronbach’s α were assessed to determine the adequacy of subscales and the allocation of an item to a subscale. Inter-item correlations between 0.2 and 0.5, item-total correlations ≥ 0.3, and a Cronbach’s α between 0.7 and 0.8 indicate an acceptable internal consistency [42]. Items that did not fit correlation criteria were deleted. Additionally, reliability was assessed using the Rasch model’s person separation index (PSI), which implies a high sensitivity of the measurement instrument with a value of ≥ 0.7 [65].

### Compilation of the questionnaire

Based on the results of the analyses the final version of the OBI-Care was compiled and then translated into English applying the forward-backward translation method [42] by two authors (AR, MD) and two English native speakers (Karin Simpson-Parker [KS-P] and Layve Dirk Roeder [LDR]). For forward translation, the OBI-Care was translated from German into English by two persons (AR and KS-P) independently. Following recommendations, one person (AR) was an expert on the construct to be measured and one person (KS-P) was an expert on the language. AR, KS-P and MD compared both translations, discussed challenging phrases and solved discrepancies. Afterwards, the combined translation was translated back into German by a native occupational therapist (LDR). Finally, AR and MD checked whether the meaning of the items stayed the same over the translation process.

### Ethical considerations

The ethics committees of the Medical University of Vienna, the City of Vienna, Lower Austria, the University of Salzburg, and of the Medical University of Graz approved the study. Participants confirmed their voluntary participation in this study with verbal and written informed consent. The study was conducted according to the principles expressed in the Declaration of Helsinki.

## Results

### Participants

A total of fourteen parents participated in the item creation process. Four parents (two mothers and fathers each) of preterm infants collaborated in the item creation process of the initial OBI-Care items. Mothers were 39 and 32 years old and had an International Standard Classification of Education (ISCED [69]) of 6 and 3. Fathers were 45 and 35 years old and had an ISCED level of 5 and 3. Cognitive debriefing was conducted with ten further parents (five mothers and fathers each). Mothers had a median age of 33 years (ranging from 21 to 42) and had ISCED levels ranging from 2 to 5. Fathers had a median age of 35 years (ranging from 28 to 52) and had ISCED levels ranging from 2 to 5. The panel of experts consisted of occupational therapists, physiotherapists, psychologists, nurses and one pediatrician of the neonatal care team of the Medical University of Vienna. Some of them are trained and skilled in questionnaire development and all of them are experts in neonatal intensive care.

A total of three hundred and eight parents of preterm infants filled in the OBI-Care. Data of 304 parents of preterm infants were used for the examination of the measurement properties of the OBI-Care. Sociodemographic characteristics of these parents are depicted in Table 1.

**Table 1:** Sociodemographic data of parents.

Parent’s characteristics		Female	Male	Total
Sex		195 (64%)	109 (36%)	304 (100%)
Mean age in years (±SD)		33 (±6)	35 (±6)	34 (±6)
Educational level ^a^				
	level 0–1	-	-	-
level 2–3		98 (50%)	60 (56%)	158 (52%)
level 4–8		91 (47%)	43 (39%)	134 (44%)
not specified		6 (3%)	6 (5%)	12 (4%)
Employment status ^b^				
	student	4 (2%)	2 (2%)	6 (2%)
parental leave		175 (90%)	4 (4%)	179 (59%)
self-employed		8 (4%)	20 (18%)	28 (9%)
employed		11 (6%)	83 (76%)	94 (31%)
unemployed		4 (2%)	2 (2%)	6 (2%)
not specified		1 (1%)	4 (4%)	5 (2%)
**Children’s characteristics**				
Mean hospital stay in days (±SD, range)		71.7 (±29.1, 18–164)		
Mean APGAR Score (±SD, range)		8.5 (±1.1, 2–10)		
Mean gestational age (±SD, range)		28+3 (± 2, 23+2–36+0)		
Mean birthweight in gram (±SD, range)		1045.4 (±286.6, 470–1500)		

^a^ = Levels of the International Standard Classification of Education (ISCED)

^b^ = multiple answers allowed

^c^ = Major groups of the International Classification of Occupations, Austria (Ö-ISCO); APGAR = Appearance, Pulse, Grimace, Activity und Respiration Score; SD = Standard deviation.

### Created set of items

Based on previously identified components, 49 items were formulated together with parents of preterm infants. For example, item *3b “In case of changed circumstances*, *such as an extended hospital stay of your relative*, *how satisfied are you with your options to spend more time on some occupations and less time on others*?*”* was created upon the component *adapt time expenditure*. Upon request by the parents, a five-choice response scale (reaching from 1 = very satisfied to 5 = very dissatisfied) was created for each item. Following the results of cognitive debriefing, some words and phrases were changed. For instance, the term “relation” was changed to “ratio” in the introductory sentence of subscale 2. Additionally, some items were supplemented with examples to improve their understandability, such as item *1c* “*How satisfied are you with the frequency and duration of occupations in the area life management (e*. *g*.: *doing administrative errands or bank transfers)*?”. *Participants of cognitive debriefing confirmed the need of a five-choice response scale*. *Thus*, response options were not revised.

### Explored measurement properties

Dimensionality testing and factor analysis of the first set of forty-nine items indicated multidimensionality of the measure. Forty-five percent of the t-tests were located outside the acceptable significance range and factor analysis indicated nine factors. Additionally, analysis with the Rasch model showed a poor fit to the Rasch model. Mean item fit was 0.38 (±2.06) and chi-square probability value *p* < 0.01. Cronbach’s α and PSI of the first set of items were 0.96 each.

Thus, out of the 49 items, nine generic and six specific items with statistical redundancy or similar content were deleted. Another nine items that asked for support, understanding and appreciation of the social environment were deleted due to their poor correlation with the construct to be measured. Three other items that showed poor item fit to the Rasch model were deleted, for example one item on personal hygiene.

Factor analysis and correlation analysis of the remaining twenty-two items displayed three factors. Thus, items were summarized in three subscales. Factor analysis for each subscale showed that each subscale consisted of one factor which indicates unidimensionality of the subscales.

The three subscales showed overall fit to the Rasch model. Despite item *1i*, all items of the questionnaire complied with criteria of an item fit. Table 2 provides an overview of the overall fit and item fit for each subscale.

**Table 2:** Construct validity and internal consistency.

**Subscale 1**	**Cronbach’s Alpha**		**PSI**	**chi-square p**	**mean item fit**
	0.89		0.88	0.03	0.18 (±1.41)
	**item statistics** ^**a**^		**fit statistics** ^**a**^		
**Items**	**location**	**SE**	**residual** *	**chi-square** ^b^^**^	**f-statistics** ^**b**^
Item_1a	0.306	0.073	0.493	2.528	0.664
Item_1b	0.184	0.069	-0.448	6.376	1.473
Item_1c	0.1	0.073	-0.535	1.907	0.662
Item_1d	-0.825	0.07	-0.384	7.437	2.432
Item_1e	0.361	0.07	0.122	0.821	0.176
Item_1f	-0.311	0.069	-0.977	6.596	2.086
Item_1g	-0.18	0.07	-1.519	9.717	3.319
Item_1h	-0.027	0.07	2.245	3.938	0.821
Item_1i	0.393	0.072	**2.663**	13.717	3.055
**Subscale 2**	**Cronbach’s Alpha**		**PSI**	**chi-square p**	**mean item fit**
	0.88		0.87	0.78	0.12 (± 1.08)
	**item statistics** ^**a**^			**fit statistics** ^**a**^	
**Items**	**location**	**SE**	**residual**	**chi-square** ^**c**^	**f-statistics** ^**c**^
Item_2a	-0.06	0.081	0.519	0.415	0.108
Item_2b	0.139	0.09	-1.168	5.684	1.909
Item_2c	-0.029	0.088	0.176	2.541	0.592
Item_2d	0.156	0.087	-1.498	5.417	2.063
Item_2e	0.103	0.082	0.291	3.151	0.885
Item_2f	0.269	0.085	1.153	0.938	0.257
Item_2g	-0.578	0.074	1.355	3.733	0.984
**Subscale 3**	**Cronbach’s Alpha**		**PSI**	**chi-square p**	**mean item fit**
	0.87		0.84	0.54	0.25 (±1.14)
	**item statistics** ^**a**^			**fit statistics** ^**a**^	
**Items**	**location**	**SE**	**residual**	**chi-square** ^**d**^	**f-statistics** ^**d**^
Item_3a	-0.056	0.084	0.932	3.084	0.784
Item_3b	0.008	0.083	-0.104	4.431	1.348
Item_3c	0.548	0.086	0.029	1.356	0.358
Item_3d	0.212	0.086	-1.094	5.081	1.68
Item_3e	-0.254	0.084	-0.403	3.602	1.095
Item_3f	-0.458	0.077	2.145	5.04	1.231

^a^ = rounded to three decimals

^b^ = Bonferroni adjusted probability level = 0.001111

^c^ = Bonferroni adjusted probability level = 0.001429)

^d^ = Bonferroni adjusted probability level = 0.001667)

* Deviations from the recommended range of -2.5 to +2.5 indicating misfit are bold

**Bonferroni adjusted statistically significant deviations indicating misfit are bold; PSI = Person Separation Index; p = probability; SE = Standard error.

Moreover, analyses showed no evidence of DIF. All items had ordered thresholds, a good degree of separation and all categories were represented in the responses (S1 Fig). Overall, the twenty-two items showed to have a good construct validity.

Subscales and the allocation of the items to their hypothesized scale were adequate and demonstrated internal consistency. Inter-item correlations for all subscales satisfied the given criteria, except item *3c* and *3d* (inter-item correlation = 0.75). Additionally, Cronbach’s α supported the suitability of the subscales (Table 2). The subscales could distinguish between persons with high or low ability (item-total correlation = 0.55 to 0.75). PSI values showed that persons with a high ability achieved a high score while persons with a low ability achieved a low score (Table 2).

The final set of items summarized in three subscales demonstrated unidimensionality, internal consistency and construct validity and thus measured occupational balance in a valid and reliable way.

### Questionnaire

The twenty-two items were compiled to the final (German) version of the OBI-Care (S1 Table), including three subscales representing the following dimensions of occupational balance: *occupational areas* (subscale 1), *occupational characteristics* (subscale 2) and *occupational* resilience (subscale 3). Subscale 1 assesses the satisfaction with meaningful activities in terms of their extent, distribution, and variety in different areas of meaningful activities. Subscale 2 explores the properties and effects of meaningful activities. Subscale 3 measures the ability to perpetuate and to find new meaningful activities accompanying changed life circumstances.

The English version of the OBI-Care is presented in S2 Table.

For calculation, raw data of the subscales are summarized into sum scores ranging from 5 to 45 for subscale 1, 5 to 35 for subscale 2 and 5 to 30 for subscale 3. Lower sum scores indicate satisfaction within the three identified dimensions of occupational balance and vice versa.

## Discussion

Within the current study a valid and reliable self-reported questionnaire on occupational balance in informal caregivers could be developed. To our knowledge, this is the first self-reported questionnaire on occupational balance that was specifically developed for and with parents of preterm infants [43].

A total of 49 items was created and subsequently reduced, following standard procedures [42]. The components of several items, such as the component *self-care* had been assessed in other occupational balance measurement instruments before [43]. However, the content of some other items, such as *adapt time expenditure* are novel and have not been considered in any occupational balance measurement instruments so far.

As expected, some items showed a poor correlation, item misfit or both and, except for three items, were therefore deleted [60, 68, 70]. We did not delete item *1i* on satisfaction with job, education and training, item *3c* and *3d* on satisfaction with knowledge gathering and skills acquisition for new occupations. These items address the components *productivity*, *knowledge gathering* and *skills acquisition* which present essential components of occupational balance but are not represented by any further items.

Analyses of the remaining items indicated a need for subscales. The need for subscales when assessing occupational balance was also found in a previous study [57]. Thus, the remaining twenty-two items were summarized into three subscales to satisfy measurement properties [71]. To our knowledge, this is the first time that subscales have been used to assess occupational balance [43].

In accordance with the three subscales of the OBI-Care, we identified three dimensions of occupational balance. Previous studies identified three dimensions of occupational balance as well [72, 73]. However, these dimensions differ partially from the dimensions of occupational balance identified in the current study. Nevertheless, a balance in *occupational areas* was already included in the conceptualization of occupational balance at the beginning. *Occupational characteristics*, *such as* a balance between demanding and relaxing occupations, between chosen and obligatory occupations and between caring for oneself and others were also described earlier [32, 43, 73–75]. To our knowledge, the dimension *occupational resilience* is novel. However, components of *occupational resilience*, such as the adaption of activities, were found to be relevant for one’s occupational balance before [43, 57]. Further studies on the conceptualization and dimensions of occupational balance are recommended.

According to previous studies, a satisfying occupational balance might have a positive effect on the health of both, caregivers and persons to be cared for [29, 31]. Another study implies that there might be a link between the engagement in meaningful occupations and parental and infant health [76]. We therefore recommend professionals working in NICUs, such as occupational therapists to support parents of preterm infants in strengthening their occupational balance. An improvement of parents’ occupational balance may have a favorable impact on the health outcomes of preterm infants, which has to be tested in future studies. The easy calculation of raw data generated with the OBI-Care supports its application in the assessment of occupational balance in clinical practice.

This study has several strengths and limitations. The creation of items together with parents of preterm infants strengthened the OBI-Care’s construct validity [46, 77]. However, the use of a user-centered design approach [51] for the development of the OBI-Care may have led to the creation of other items. The application of the Rasch model facilitated the identification of a set of items that measures occupational balance in a valid and reliable way and ensures further analyses on interval scale level. Additionally, the recruitment of participants from different NICUs in Austria led to a high diversity within the sample and to the achievement of a sample size larger than expected. Nonetheless, further studies are needed to evaluate the application of the OBI-Care in different settings.

Although the translation of the German version of the OBI-Care into English followed standard procedures [42], we did not validate the English version of the OBI-Care. Thus, a validation of the English version is required. Furthermore, to ensure cross-cultural validity, the examination of the measurement properties of the OBI-Care in different populations is warranted [42]. Moreover, we did not aim to determine cutoff values within this study. Hence, further studies are needed to define cut-off values of occupational balance to determine a need for and to evaluate occupational balance interventions in neonatal intensive care.

## Conclusions

The OBI-Care was successfully developed to assess occupational therapy in informal caregivers, such as parents of preterm infants, and demonstrated construct validity and internal consistency. As a result, the OBI-Care might be helpful for health professionals working in NICUs to assess occupational balance of parents of preterm infants and to adapt their treatment.

## Supporting information

S1 Fig Threshold ordering. Ordered thresholds indicate that the item’s response categories operate appropriate.
(DOCX)

S1 Table German version of the occupational balance in informal caregivers (OBI-Care). (DOCX)

S2 Table Occupational balance in informal caregivers (OBI-Care). (DOCX)
