# Supplementary figures and images for: Development and validation of a self-reported questionnaire to assess occupational balance in parents of preterm infants
Source: PLoS One. 2021 Nov 15;16(11):e0259648. doi: 10.1371/journal.pone.0259648 (PMC8592439; doi:10.1371/journal.pone.0259648)

S1 Fig. Threshold ordering


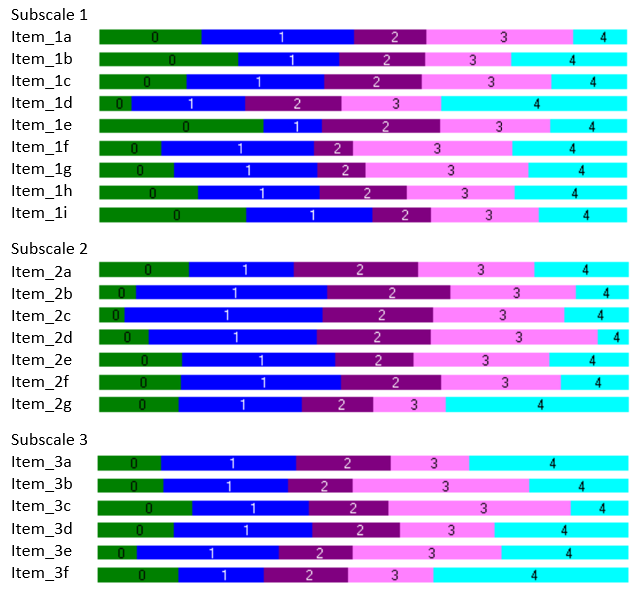

Supplement: S1 Fig — Ordered thresholds indicate that the item’s response categories operate appropriate. (DOCX) [file pone.0259648.s001.docx]
