# Supplementary material for: Development and validation of a self-reported questionnaire to assess occupational balance in parents of preterm infants
Source: PLoS One. 2021 Nov 15;16(11):e0259648. doi: 10.1371/journal.pone.0259648 (PMC8592439; doi:10.1371/journal.pone.0259648)
Supplement: S1 Table — (DOCX) [file pone.0259648.s002.docx]

S1 Table. German version of the Occupational Balance in informal Caregivers (OBI-Care)

| **Fragebogen zur Betätigungsbalance bei pflegenden Angehörigen**  ©Dür, M., Brückner, V., Fuiko, R., Leeb, C., Röschel, A. und Berger, A. (2019) | | | | | | | | | | | |  |
| --- | --- | --- | --- | --- | --- | --- | --- | --- | --- | --- | --- | --- |
| „Betätigung“ oder „Tätigkeit“ bezieht sich auf alles was Sie tun, tun wollen oder getan haben, einschließlich einfacher Tätigkeiten, wie z.B. sich selbst waschen oder anziehen. Das können Tätigkeiten in Ihrem Job, Freizeit- und Erholungstätigkeiten (z.B. Musik hören oder schlafen), Tätigkeiten im Haushalt, die Betreuung von Kindern und Enkelkindern, als auch die Pflege von Angehörigen sein. Die Betätigungsbalance ist eine als gut und befriedigend erlebte Mischung von verschiedenen Tätigkeiten. Bitte kreuzen Sie pro Frage immer jene Möglichkeit an, die am ehesten auf Sie zutrifft. | | | | | | | | | | | |  |
| **Bitte denken Sie an die Häufigkeit und Dauer Ihrer Tätigkeiten.** | | | | | | | | | | | |  |
| **1. Wie zufrieden sind Sie derzeit mit dem Ausmaß an Tätigkeiten in den folgenden Bereichen …** | | Sehr zufrieden | | Eher  zufrieden | | Teils zufrieden, teils unzufrieden | | Eher unzufrieden | | Sehr unzufrieden | |  |
| a | Haushalt? (z.B.: Wäsche waschen) | |  | |  | |  | |  | |  | |
| b | die (private) Versorgung anderer? (z.B.: für die Familie kochen) | |  | |  | |  | |  | |  | |
| c | Lebensmanagement? (z.B.: Behörden- und Bank Wege) | |  | |  | |  | |  | |  | |
| d | körperliche Bewegung/Sport? (z.B.: spazieren gehen) | |  | |  | |  | |  | |  | |
| e | soziale Kontakte? (z.B.: Familie, Freunde, KollegInnen) | |  | |  | |  | |  | |  | |
| f | Gesundheit und Wohlbefinden? (z.B.: sich massieren lassen) | |  | |  | |  | |  | |  | |
| g | Freizeit? (z.B.: lesen) | |  | |  | |  | |  | |  | |
| h | Schlaf? | |  | |  | |  | |  | |  | |
| i | Job, Fort- und Weiterbildung? (Bitte auch ankreuzen, wenn Sie derzeit nicht erwerbstätig oder in Fort- und Weiterbildung sind.) | |  | |  | |  | |  | |  | |

***To be continued***

Continuation S1 Table.

| **Fortsetzung: Fragebogen zur Betätigungsbalance bei pflegenden Angehörigen**  ©Dür, M., Brückner, V., Fuiko, R., Leeb, C., Röschel, A. und Berger, A. (2019) | | | | | | |
| --- | --- | --- | --- | --- | --- | --- |
| **Bitte denken Sie an die unterschiedlichen Eigenschaften und Wirkungsweisen Ihrer Tätigkeiten.** | | | | | | |
| **2.** | **Wie zufrieden sind Sie derzeit mit dem Verhältnis von…** | Sehr zufrieden | Eher  zufrieden | Teils zufrieden, teils unzufrieden | Eher unzufrieden | Sehr unzufrieden |
| a | Tätigkeiten, die Sie von sich aus tun, und jenen, die Sie aufgrund anderer tun? (z.B.: einem Hobby nachgehen vs. Sport betreiben auf Anraten von ÄrztInnen) |  |  |  |  |  |
| b | gewöhnlichen und ungewöhnlichen Tagesabläufen? (z.B.: Tage, an denen Sie dasselbe in derselben Reihenfolge wie immer tun vs. Tage an denen Sie etwas ganz anderes oder in einer ganz anderen Reihenfolge tun) |  |  |  |  |  |
| c | vorhersehbaren und unvorhersehbaren Tätigkeiten? (z.B.: begleitende Tätigkeiten bei geplanter vs. Notaufnahme der Angehörigen) |  |  |  |  |  |
| d | wichtigen und weniger wichtigen Tätigkeiten? (z.B.: sich zum Ausgleich bewegen vs. Zuhause aufräumen) |  |  |  |  |  |
| e | körperlich anstrengenden und weniger anstrengenden Tätigkeiten? (z.B.: Gartenarbeit vs. einkaufen gehen) |  |  |  |  |  |
| f | geistig anstrengenden und weniger anstrengenden Tätigkeiten? (z.B.: Benutzung eines neuen Computerprogrammes vs. Schreiben eines Emails) |  |  |  |  |  |
| g | Tätigkeiten bei denen Sie drinnen und draußen tätig sind? (z.B.: Büro oder Wohnraum vs. Grünanlagen) |  |  |  |  |  |

***To be continued***

Continuation S1 Table.

| **Fortsetzung: Fragebogen zur Betätigungsbalance bei pflegenden Angehörigen**  ©Dür, M., Brückner, V., Fuiko, R., Leeb, C., Röschel, A. und Berger, A. (2019) | | | | | | |
| --- | --- | --- | --- | --- | --- | --- |
| **Bitte denken Sie an die Möglichkeiten Ihre Tätigkeiten auf veränderte Lebensumstände, wie zum Beispiel einen längeren Krankenhausaufenthalt ihrer/ihres Angehörigen, abzustimmen.** | | | | | | |
| **3. Wie zufrieden sind Sie derzeit mit Ihren Möglichkeiten…** | | Sehr zufrieden | Eher  zufrieden | Teils zufrieden, teils unzufrieden | Eher unzufrieden | Sehr unzufrieden |
| a | aufgrund veränderter Lebensumstände die Reihenfolge Ihrer Tätigkeiten zu verändern? |  |  |  |  |  |
| b | aufgrund veränderter Lebensumstände für bestimmte Tätigkeiten mehr Zeit und für andere weniger Zeit aufzuwenden? |  |  |  |  |  |
| c | Information für die Durchführung neuer Tätigkeiten einzuholen? |  |  |  |  |  |
| d | Fähigkeiten für die Durchführung neuer Tätigkeiten zu erwerben? |  |  |  |  |  |
| e | Tätigkeiten, die für Sie sehr wichtig sind, weiterhin ausführen zu können? |  |  |  |  |  |
| f | neue Tätigkeiten zu finden, die Ihnen etwas bedeuten? |  |  |  |  |  |
