# Supplementary material for: Development and validation of a self-reported questionnaire to assess occupational balance in parents of preterm infants
Source: PLoS One. 2021 Nov 15;16(11):e0259648. doi: 10.1371/journal.pone.0259648 (PMC8592439; doi:10.1371/journal.pone.0259648)
Supplement: S2 Table — (DOCX) [file pone.0259648.s003.docx]

S2 Table. Occupational Balance in Informal Caregivers (OBI-Care)

| **Occupational Balance in Informal Caregivers (OBI-Care)**  ©Dür, M., Brückner, V., Fuiko, R., Leeb, C., Röschel, A. and Berger, A. (2019) | | | | | | |
| --- | --- | --- | --- | --- | --- | --- |
| “Occupation” refers to anything you do, want to do or did in the past. This might be very simple activities such as bathing or getting dressed, job related activities, leisure and relaxation activities, household activities as well as childcare and the support and care of relatives. Occupational balance describes the individual perception of having the right amount and variation of different occupations. Please tick the most applicable option for each question. | | | | | | |
| **Please think about the extent of your occupations.** | | | | | | |
| **1. How satisfied are you with the frequency and duration of occupations in the following areas …** | | very satisfied | satisfied | partly satisfied/ dissatisfied | dissatisfied | very dissatisfied |
| a | household? (e.g.: doing the laundry) |  |  |  |  |  |
| b | caring for others? (e.g.: cooking for your family) |  |  |  |  |  |
| c | life management (e.g.: administrative errands or bank transfers) |  |  |  |  |  |
| d | physical activity / sports (e.g.: going for a walk) |  |  |  |  |  |
| e | social contacts? (e.g.: spending time with family, friends or colleagues) |  |  |  |  |  |
| f | health and well-being? (e.g.: getting a massage) |  |  |  |  |  |
| g | leisure? (e.g.: reading a book) |  |  |  |  |  |
| h | sleep? |  |  |  |  |  |
| i | job, further education, and training? (Please also choose an option if you are not currently employed, in further education or training) |  |  |  |  |  |

***To be continued***

Continuation S2 Table.

| **Continuation: Occupational Balance in Informal Caregivers (OBI-Care)**  ©Dür, M., Brückner, V., Fuiko, R., Leeb, C., Röschel, A. and Berger, A. (2019) | | | | | | |
| --- | --- | --- | --- | --- | --- | --- |
| **Please think about the different characteristics and effects of your occupations.** | | | | | | |
| **2. How satisfied are you with the ratio of …** | | very satisfied | satisfied | partly satisfied/ dissatisfied | dissatisfied | very dissatisfied |
| a | occupations you do on your own initiative and those you do because of others? (e.g.: pursuing a hobby vs. exercising on the advice of doctors) |  |  |  |  |  |
| b | usual and unusual daily routines? (e.g.: days when you do the same things in the same order as usual vs. days when you do something completely different or in a completely different order) |  |  |  |  |  |
| c | predictable and unpredictable occupations? (e.g.: occupations associated with planned vs. emergency hospital treatments) |  |  |  |  |  |
| d | important and less important occupations? (e.g.: exercise to maintain one’s well-being vs. tidying up) |  |  |  |  |  |
| e | physically demanding and less physically demanding occupations? (e.g.: gardening vs. doing the shopping) |  |  |  |  |  |
| f | mentally demanding and less mentally demanding occupations? (e.g.: using a new computer programme vs. writing an e-mail) |  |  |  |  |  |
| g | indoor and outdoor occupations? (e.g.: office or living space vs. parks or green areas) |  |  |  |  |  |

***To be continued***

Continuation S2 Table.

| **Continuation: Occupational Balance in Informal Caregivers (OBI-Care)**  ©Dür, M., Brückner, V., Fuiko, R., Leeb, C., Röschel, A. and Berger, A. (2019) | | | | | | |
| --- | --- | --- | --- | --- | --- | --- |
| **Please think about the adaptability of your occupations.** | |  | | | | |
| **3. In case of changed circumstances, such as an extended hospital stay of your relative, how satisfied are you with your options to …** | | very satisfied | satisfied | partly satisfied/ dissatisfied | dissatisfied | very dissatisfied |
| a | change the order of your occupations? |  |  |  |  |  |
| b | spend more time on some occupations and less time on others? |  |  |  |  |  |
| c | gather required information to perform new occupations? |  |  |  |  |  |
| d | develop required skills to perform new occupations? |  |  |  |  |  |
| e | continue to pursue occupations that are meaningful to you? |  |  |  |  |  |
| f | find new occupations that are meaningful to you? |  |  |  |  |  |
